# Social Functioning Trajectories of Young First-Episode Psychosis Patients with and without Cannabis Misuse: A 30-Month Follow-Up Study

**DOI:** 10.1371/journal.pone.0122404

**Published:** 2015-04-07

**Authors:** César González-Blanch, John F. Gleeson, Peter Koval, Sue M. Cotton, Patrick D. McGorry, Mario Alvarez-Jimenez

**Affiliations:** 1 Mental Health Centre, University Hospital “Marqués de Valdecilla”, Santander, Spain; 2 School of Psychology, Australian Catholic University, Melbourne, Australia; 3 Faculty of Psychology and Educational Sciences, KU Leuven, Leuven, Belgium; 4 Centre for Youth Mental Health, The University of Melbourne, Melbourne, Australia; 5 Orygen Youth Health Research Centre. Melbourne, Australia; Benito Menni Complejo Asistencial en Salud Mental, SPAIN

## Abstract

The aim of the study was to investigate trajectories of social functioning in young people with first-episode psychosis (FEP) with and without cannabis misuse using a secondary analysis of data from the Episode-II trial. Forty-two young people with FEP and comorbid cannabis use disorder were compared with 39 young people with FEP but without a cannabis use disorder. Social functioning was assessed every 6 months during a 30-month follow-up. Multilevel linear growth curve modeling was used to compare the social functioning trajectories over time for those with and without cannabis misuse. Cannabis misuse was not associated with social functioning at baseline assessment. Over a 30-month follow-up, FEP patients without cannabis disorder showed significant improvements in their social functioning, whereas patients with cannabis misuse at baseline displayed no such improvement. Patients with and without cannabis misuse differed significantly in their levels of social functioning after 24 months. Similar results were obtained after adjusting for potential confounders (i.e., age, gender, negative symptoms, premorbid functioning, DSM-IV diagnoses, baseline social functioning and other substance use). In the context of a specialized early intervention service, patients with cannabis misuse at baseline did not attain the improvements in social outcomes observed in their counterparts without cannabis misuse. There is a need to develop effective interventions to reduce cannabis misuse to ultimately improve social outcomes in young people with psychosis.

## Introduction

Cannabis is the most widely used illicit substance in the world: there are about 180 million cannabis users worldwide [1]. People with psychotic disorders have higher rates of cannabis use compared with the population at large [2]. As a result, cannabis use disorders are common in schizophrenia patients, particularly in younger and first-episode patient samples [3].

While systematic reviews of cannabis use in psychosis have shown that people who have used cannabis have an increase in incidence of psychosis of about 40% [4], the impact of cannabis use on the outcome of patients already suffering from psychotic disorders remains unclear. The use of cannabis is fairly consistently associated with increased risk of relapse or rehospitalisation and with decreased treatment adherence. However, evidence for associations with other treatment outcomes is rather equivocal [5].

Although social dysfunction is a core feature of psychotic disorders, little attention has been paid until recently to the impact of cannabis use on social impairments. The few longitudinal studies that have examined this relationship in psychotic disorders have largely supported the notion that cannabis use has a detrimental effect on social outcomes [6–9]. However, some studies have not found this association [10,11], and some have found that social outcome was predicted by changes in cannabis use over time [12,13] or that negative social outcomes were associated with persistent cannabis use rather than with baseline status [14]. These divergent findings may be explained by methodological differences such as disparity in the samples, the time between assessments, or in the covariates used to control for potential confounders (e.g., illness severity or baseline social functioning). It has been recommended that research in this realm should have a longitudinal design, with repeated measures as well as adjustment for baseline levels of the outcome measure and other relevant confounding variables [5].

The aim of this study was to examine the predictive value of cannabis misuse at baseline on social functioning, and the potentially different social functioning trajectories of those with cannabis misuse compared to those with no cannabis misuse in a sample of young remitted FEP patients over a period of 30 months.

## Method

### Participants

In the present study we analysed data from all 81 participants in the Episode II trial as one cohort. The Episode II trial compared Treatment as Usual (TAU) with a combined family and individual Relapse Prevention Treatment (RPT) plus TAU within two specialist FEP services. There were 6 assessment time points: baseline, 7, 12, 18, 24, and 30 months [15–18]. Patients from the Early Psychosis Prevention and Intervention Centre (EPPIC) in Melbourne and from JIGSAW, Barwon Health in Geelong, Victoria, Australia, were recruited between November 2003 and May 2005. The study inclusion criteria were a diagnosis of a first episode of a Diagnostic and Statistical Manual of Mental Disorders, Fourth Edition—Text Revision (DSM-IV-TR) psychotic disorder [19], less than 6 months of prior treatment with antipsychotic medications, age 15–25 years inclusive, and remission on positive symptoms of psychosis. Remission was defined as 4 weeks or more of scores of 3 (mild) or below on the subscale items hallucinations, unusual thought disorder, conceptual disorganization, and suspiciousness on the expanded version of the Brief Psychiatric Rating Scale [BPRS; 20]. Exclusion criteria were ongoing active positive psychotic symptoms, severe intellectual disability, inability to converse in or read English, and participation in previous CBT trials.

### Ethics Statement

The study was approved by the Northwestern Mental Health and the Barwon Health Research and Ethics Committees. All patient participants provided written informed consent.

### Assessment

The Structured Clinical Interview for DSM-IV (SCID), including psychoses, substance dependence and abuse, mood disorders and personality disorders was completed at baseline [21]. Symptom measures included the Montgomery–Åsberg Depression Rating Scale [MADRS; 22], which provides a measure of the severity of depressive symptoms; the BPRS, which provides severity ratings across a broad range of psychotic and non-psychotic symptoms; and the Scale for the Assessment of Negative Symptoms [SANS; 23], a measure of negative symptoms. The Pre-morbid Adjustment Scale (PAS) was used to evaluate pre-morbid functioning [24]. To focus on early adjustment, we created an average score for social and academic domains based only on the first three areas of development (i.e. childhood, early adolescence, late adolescence). Full-Scale IQ (FSIQ) was estimated based on the performance on the Wechsler Test of Adult Reading (WTAR) [25].

Social functioning was assessed using the Social and Occupational Functioning Assessment Scale (SOFAS [26]). The SOFAS was developed as an additional AXIS V clinician-rated measure of global social and occupational functioning, and is not directly influenced by the overall severity of the individual's psychological symptoms. The SOFAS yields an overall score of current functioning ranging from 0 to 100, with lower scores representing lower functioning. This scale has been used as a measure of functional outcome in FEP studies [27].

Cannabis misuse was defined as meeting DSM-IV-TR criteria for cannabis abuse or dependence at baseline assessment which was assessed by the SCID. Problematic cannabis use at each subsequent time point was determined using the Alcohol, Smoking and Substance Involvement Screening Test (ASSIST [28]). The ASSIST is a valid screening test for psychoactive substances in individuals who use a number of different substances and a valid measure of severity of dependence for the substance that is most problematic for the person concerned. The validity of this scale has been proven across different cultures, including the Australian context [29]. A cut-off score of ≥ 2 has been demonstrated to have the best sensitivity and specificity for DSM-IV cannabis use disorder among people with FEP [30]. Using baseline data of this study, a cutoff score in the ASSIST set at ≥ 2 had a sensitivity of 83% and a specificity of 87% for the diagnosis of cannabis disorder [31]. Sensitivity and specificity values above 0.8 are considered optimal for screening tools [32].

### Statistical analyses

To compare patients with and without cannabis misuse at each assessment, we used Student's t-test and the chi-square test, as appropriate. In the instance of notably skewed distributions, logarithmic or square root transformations were performed. Analyses were performed using SPSS − version 18.0 (SPSS Inc).

To compare change trajectories of social functioning over time for each cannabis group, we used multilevel linear growth curve modeling [33], estimated using HLM 7.01 (SSI Inc.). Thus, measurement occasion (i.e., time) was a within-person predictor and baseline cannabis disorder diagnosis was a between-person predictor. The Level-1 (within-person) model equation was as follows:

$$SOFAS_{ti}=\pi_{0i}+\pi_{1i}*\left(Time_{ti}\right)+e_{ti}$$

Here, a person *i*’s SOFAS score at assessment *t* is modeled as a function of an intercept (π_0*i*_) and a slope (π_1*i*_) representing the linear effect of time (Time_*ti*_: 0 = baseline, 5 = 30 months, with equal intervals for each intermediate follow-up). Thus, the Level-1 intercept (π_0*i*_) reflects person *i*’s baseline level of social functioning (i.e., SOFAS at Time_*ti*_ = 0), whereas the Level-1 slope (π_1*i*_) reflects person *i*'s rate of change in SOFAS over 30 months. The intercept and slope were allowed to vary randomly across persons and modeled as a function of cannabis disorder group at Level-2, as follows:

$$\begin{array}{l} \pi_{0i}=\beta_{00}+\beta_{01}\left(Cannabis\;Misuse_{i}\right)+r_{0i}\\ {\text{π}}_{1i}=\beta_{10}+\beta_{11}\left(Cannabis\;Misuse_{i}\right)+r_{1i} \end{array}$$

Here, the Level-1 intercept and slope were modeled as function of a dummy variable, *Cannabis Misuse*_*i*_ (1 = cannabis misuse; 0 = no cannabis misuse), indicating whether person *i* was diagnosed with a DSM-IV cannabis use disorder at baseline. Thus, β_00_ is an estimate of SOFAS at baseline among FEP patients without cannabis disorder, whereas β_01_ represents how much patients with cannabis disorder differ from those without cannabis disorder in terms of baseline SOFAS. Similarly, β_10_ is an estimate of the rate of change in SOFAS over the 30-month follow-up among individuals without cannabis misuse disorder, whereas β_11_ reflects the difference in SOFAS change trajectory among patients with a cannabis misuse disorder.

## Results

### Descriptive statistics and group differences in baseline characteristics

Forty-two (51.9%) patients had a baseline DSM-IV diagnosis of cannabis use disorder (abuse or dependence). Demographic and other baseline characteristics for the entire sample and separately by cannabis disorder group are presented in Table 1. As shown in Table 1, the cannabis groups differed on a number of baseline measures. Specifically, relative to patients without cannabis misuse, a (marginally) significantly higher proportion of patients with cannabis disorder were: a) assigned to the RPT condition (*p* = .096); b) diagnosed with schizophrenia (*p* = .005), bipolar disorder (*p* = .048), or substance-induced psychotic disorder (*p* = .089); and c) diagnosed with amphetamine misuse (*p* = .003), hallucinogen misuse (*p* = .003), or cocaine misuse (*p* = .089). In contrast, cannabis disorder patients were less likely to be diagnosed with psychotic disorder not otherwise specified (*p* = .030), or a major depressive episode with psychotic features (*p* = .059). Finally, patients in the cannabis misuse group were taking significantly higher doses, calculated using chlorpromazine (CPZ) equivalents, of antipsychotic medication (*p* <. 020) and were significantly older than patients without a baseline cannabis disorder diagnosis (*p* = .032). There were no other group differences on demographics, diagnoses, or measures of baseline functioning.

**Table 1:** Demographic and baseline clinical characteristics of the total sample and by cannabis misuse group.

	**Total Sample (*N* = 81)**		**Cannabis misuse (*n* = 42)**		**No Cannabis misuse (*n* = 39)**		
Dichotomous variables	***n***	**%**	***n***	**%**	***n***	**%**	**Test of group difference**
Treatment group (RPT)	41	50.6	25	59.5	16	41.0	χ2(1, *N* = 81) = 2.77, *p* = .096
Gender (male)	51	63.0	30	71.4	21	53.8	χ2(1, *N* = 81) = 2.68, *p* = .102
Employment status (unemployed)	35	43.2	20	47.6	15	38.4	χ2(1, *N* = 81) = 0.69, *p* = .406
Psychotic disorder diagnosis							
Schizophrenia	27	33.3	20	47.6	7	17.9	χ2(1, *N* = 81) = 8.01, *p* = .005
Schizophreniform disorder	9	11.1	3	7.1	6	15.4	χ2(1, *N* = 81) = 1.39, *p* = .238
Schizoaffective disorder	4	4.9	1	2.4	3	7.7	χ2(1, *N* = 81) = 1.22, *p* = .270
Major depression with psychotic features	9	11.1	2	4.8	7	17.9	χ2(1, *N* = 81) = 3.56, *p* = .059
Bipolar disorder	4	4.9	4	9.5	0	0.0	χ2(1, *N* = 81) = 3.91, *p* = .048
Delusional disorder	1	1.2	1	2.4	0	0.0	χ2(1, *N* = 81) = 0.94, *p* = .332
Substance-induced psychotic disorder	3	3.7	3	7.1	0	0.0	χ2(1, *N* = 81) = 2.89, *p* = .089
Psychotic disorder not otherwise specified	24	29.6	8	19.0	16	41.0	χ2(1, *N* = 81) = 4.69, *p* = .030
Other substance use diagnosis							
Alcohol	20	24.7	12	28.6	8	20.5	χ2(1, *N* = 81) = 0.71, *p* = .401
Cocaine	3	3.7	3	7.1	0	0.0	χ2(1, *N* = 81) = 2.89, *p* = .089
Opioid	6	7.4	4	9.5	2	5.1	χ2(1, *N* = 81) = 0.57, *p* = .450
Amphetamine	15	18.5	13	31.0	2	5.1	χ2(1, *N* = 81) = 8.94, *p* = .003
Hallucinogen	12	14.8	11	26.2	1	2.6	χ2(1, *N* = 81) = 8.95, *p* = .003
Inhalant	2	2.5	2	4.8	0	0.0	χ2(1, *N* = 81) = 1.90, *p* = .168
Sedative, hypnotic or anxiolytic	1	1.2	1	2.4	0	0.0	χ2(1, *N* = 81) = 0.94, *p* = .332
Polysubstance	2	2.5	1	2.4	1	2.6	χ2(1, *N* = 81) = 0.00, *p* = .985
Other	1	1.2	1	2.4	0	0.0	χ2(1, *N* = 81) = 0.94, *p* = .332
Continuous variables	***M***	***SD***	***M***	***SD***	***M***	***SD***	**Test of group difference**
Age	20.11	3.05	20.81	2.53	19.36	3.40	*t*(69.99) = 2.17, *p* = .0.34^a^
Years of education	12.06	1.77	12.14	1.62	11.97	1.94	*t*(79) = 0.43, *p* = .671
FSIQ^b^ ^,^ ^e^	98.19	8.91	98.63	7.86	97.71	10.01	*t*(71) = 0.44, *p* = .663
PAS (average)^b^	0.29	0.16	0.30	0.16	0.28	0.17	*t*(62) = 0.59, *p* = .557
DUP^b^ ^,^ ^d^	384.81	567.95	418.49	630.71	349.09	500.16	*t*(66) = 0.01, *p* = .991^c^
BPRS	34.81	7.48	35.67	7.62	33.90	7.32	*t*(79) = 1.12, *p* = .265^c^
SANS	4.60	3.49	4.40	3.61	4.82	3.39	*t*(79) = -0.53, *p* = .596
MADRS	10.43	9.13	11.43	10.14	9.36	7.88	*t*(79) = 1.02, *p* = .311
SOFAS	63.17	15.89	63.69	17.50	62.62	14.17	*t*(79) = 0.30, *p* = .763
Antipsychotic dosage (CPZ equivalents)	382.35	285.53	446.26	348.56	299.27	139.06	*t*(52.41) = 2.397, *p* = .020^a^

*Note*: DUP, Duration of Untreated Psychosis; FSIQ, Full Scale IQ (see the text for tests abbreviations).

^a^ *t*-test reported with correction for unequal group variances

^b^ Due to missing data, DUP *n* = 68 (35 & 33 per group); premorbid IQ *n* = 73 (38 & 35 per group); PAS *n* = 64 (36 & 28 per group);

^c^ *t*-test based on logarithmic-transformed data due to the skewness of the raw data.

^d^ Estimated on the basis of time between onset of symptoms and entry into the service.

^e^ Estimated by the Wechsler Test of Adult Reading.

### Group differences in social functioning (SOFAS) at each assessment

**Fig 1:**
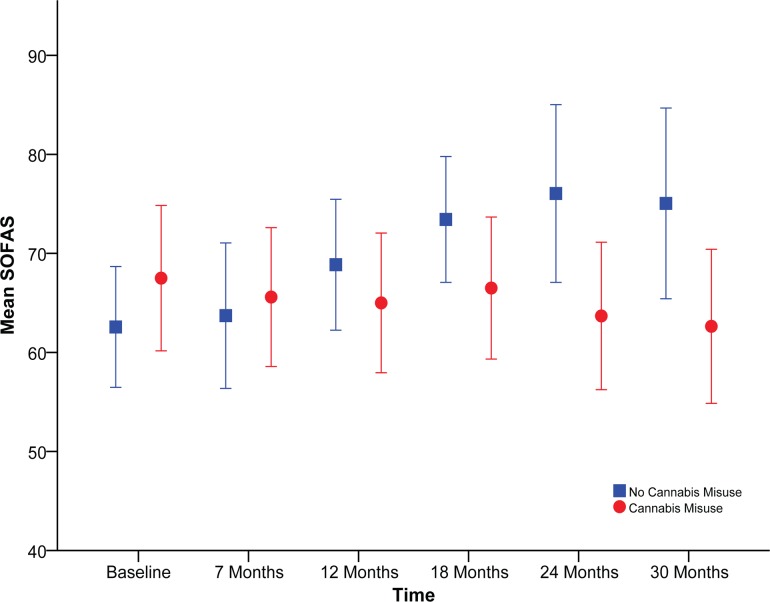
Mean social functioning (SOFAS) scores by cannabis group at each time-point. Error bars represent 95% Confidence Intervals. Differences between cannabis disorder groups at 24 and 30 months are statistically significant at *p* <.05.

Fig 1 displays mean SOFAS scores (and 95% Confidence Intervals) for FEP patients with and without comorbid cannabis disorder diagnosis at each assessment from baseline to 30-month follow-up. Patients with and without cannabis disorder did not differ significantly in their social functioning at baseline (*p* = .763; see Table 1), or at the 6-month, 12-month, or 18-month follow-ups (*p*s >. 12). However, after 24 months, patients with a baseline diagnosis of cannabis disorder had significantly lower social functioning than those without cannabis disorder, *M*_diff_ = −12.17; *SE*_diff_ = 4.80, *t*(56) = −2.54, *p* = .014, and this difference remained significant at 30-month follow-up, *M*_diff_ = −11.09; *SE*_diff_ = 5.21, *t*(51) = −2.13, *p* = .038 (see Fig 1). These results suggest that FEP patients with and without cannabis disorder showed different trajectories of social functioning over 30 months.

### Group differences in social functioning (SOFAS) change trajectories over the 30-month follow-up

**Fig 2:**
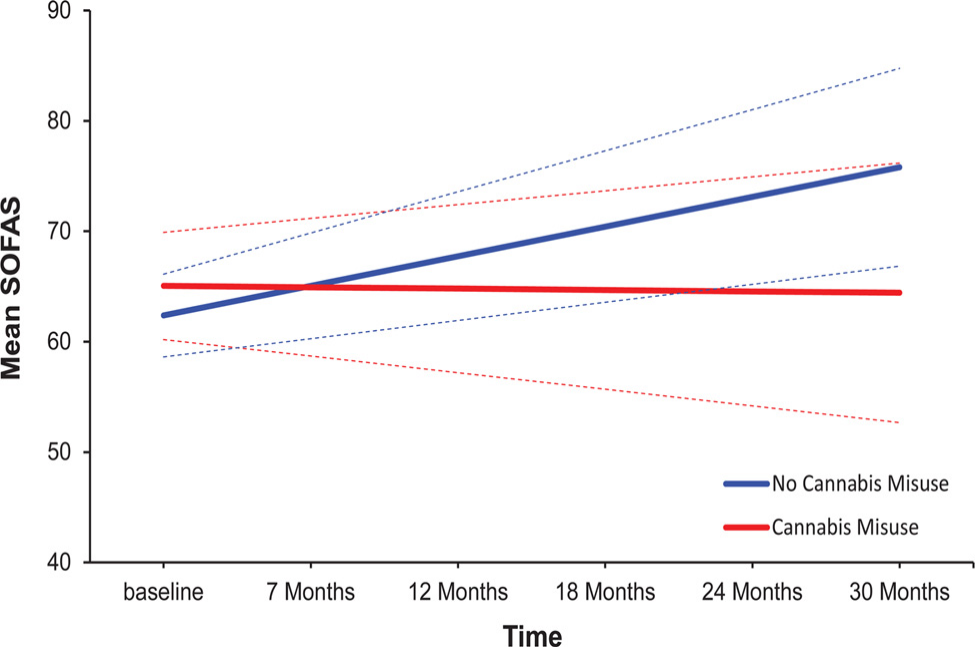
Model predicted change trajectories of social functioning (SOFAS) from baseline to 30 months by cannabis group. Dashed lines represent 95% Confidence Intervals.

To directly compare the trajectories of social functioning over the 30-month follow-up for patients with and without a comorbid cannabis misuse, we used a multilevel linear growth curve model (see above). Results of this analysis are displayed in Table 2, and revealed no effect of cannabis misuse on the intercept (*p* = .387), indicating that cannabis misuse was not associated with social functioning (SOFAS) at baseline (see also *t*-test in Table 1). However, cannabis disorder was significantly negatively related to the slope of time (*p* = .002), indicating that patients with cannabis misuse showed lesser change in social functioning over time (see Table 2, Model 1). A further simple slopes analysis revealed that whereas a significant improvement in social functioning over time was observed among patients without cannabis misuse, B = 2.68, *t*(79) = 5.14, *p* <. 001, 95% CI [1.65, 3.72], patients diagnosed with cannabis misuse showed no significant change in their level of social functioning over the 30-month follow-up, B = –0.12, *t*(79) = –0.18, *p* = .858, 95% CI [–1. 50, 1.25]. Fig 2 displays the simple slopes (with 95% CIs) reflecting change in social functioning over time for each group.

**Table 2:** Results of Separate Multilevel Linear Growth Curve Models Predicting Change in Social Functioning over 30-Months from Cannabis Misuse and other Baseline Measures Individually.

			95% CI		
	Predictor / Parameter	Estimate (*SE*)	LL	UL	*p*
*Model 1*	Cannabis misuse				
	Effect on intercept (β_01_)	2.68 (3.08)	–3.45	8.81	.387
	Effect on slope (β_11_)	–2.81 (0.87)	–4.54	–1.08	.002
*Model 2*	Treatment group				
	Effect on intercept (β_01_)	–2.64 (3.12)	–8.85	3.57	.400
	Effect on slope (β_11_)	0.06 (0.96)	–1.85	1.98	.946
*Model 3*	Schizophrenia				
	Effect on intercept (β_01_)	–10.12 (2.95)	–16.00	–4.25	<. 001
	Effect on slope (β_11_)	–1.03 (1.02)	–3.07	1.00	.315
*Model 4*	MDEP				
	Effect on intercept (β_01_)	0.64 (4.30)	–7.92	9.20	.882
	Effect on slope (β_11_)	0.92 (1.13)	–1.33	3.17	.417
*Model 5*	Bipolar disorder				
	Effect on intercept (β_01_)	11.72 (3.29)	5.17	18.27	<. 001
	Effect on slope (β_11_)	–1.80 (0.74)	–3.28	–0.33	.017
*Model 6*	SIPD				
	Effect on intercept (β_01_)	2.34 (12.09)	–21.73	26.41	.847
	Effect on slope (β_11_)	–0.91 (0.98)	–2.86	1.03	.353
*Model 7*	PDNOS				
	Effect on intercept (β_01_)	0.02 (3.42)	–6.79	6.84	.995
	Effect on slope (β_11_)	0.73 (1.12)	–1.50	2.96	.516
*Model 8*	Cocaine misuse				
	Effect on intercept (β_01_)	10.52 (5.52)	–0.47	21.51	.060
	Effect on slope (β_11_)	–3.82 (1.93)	–7.67	0.02	.051
*Model 9*	Amphetamine misuse				
	Effect on intercept (β_01_)	1.78 (4.94)	–8.05	11.62	.719
	Effect on slope (β_11_)	–0.16 (1.20)	–2.54	2.22	.895
*Model 10*	Hallucinogen misuse				
	Effect on intercept (β_01_)	–2.59 (4.54)	–11.63	6.46	.571
	Effect on slope (β_11_)	–1.86 (1.59)	–5.02	1.30	.246
*Model 11*	Age				
	Effect on intercept (β_01_)	–0.62 (0.46)	–1.54	0.30	.184
	Effect on slope (β_11_)	–0.10 (0.14)	–0.38	0.18	.479
*Model 11*	Antipsychotic dosage				
	Effect on intercept (β_01_)	–8.32 (6.01)	–20.31	3.67	.171
	Effect on slope (β_11_)	–1.84 (1.73)	–5.30	1.62	.293

*Note*.

MDEP = major depressive episode with psychotic features.

SIPD = substance-induced psychotic disorder.

PDNOS = psychotic disorder not otherwise specified.

Approx. *df* = 79 for all models except antipsychotic dosage, where *df* = 67 due to missing data

According to the ASSIST scale, 14 (17.3%) subjects changed their pattern of cannabis misuse over the 30 months follow up. Eleven of those 14 subjects changed from cannabis misuse to no cannabis misuse (i.e., their ASSIST− cannabis score was ≥ 2 at baseline but decreased to < 2 at 30-month follow-up). The remaining three subjects, who did not meet criteria for cannabis misuse (based on DSM criteria) at baseline, were classified as problematic users (based on ASSIST − cannabis scale) at 30 months follow-up. The small sample size for these subgroups precludes a separate statistical analysis. However, the main analyses were rerun without these 14 subjects and similar results were obtained to those reported above: patients without cannabis disorder showed significant improvement over time in SOFAS, B = 2.84, t(37) = 4.52, *p* <. 001, 95% CI [1.57, 4.12], whereas patients diagnosed with comorbid cannabis disorder at baseline showed no significant change in social functioning over the course of the 30-month follow-up, B = –0.96, t(37) = –1.02, *p* = .315, 95% CI [–2.88, 0.95].

### Controlling for possible confounding variables

Cannabis misuse was associated with other baseline variables (see Table 1), which may themselves have been related to changes in social functioning over time and could therefore account for the effect of cannabis misuse. To explore this, we ran additional multilevel growth curve analyses with each baseline variable that was associated with cannabis misuse at *p* <. 10 (see Table 1). These models were identical to the first multilevel growth curve model (see model equations, above). However, at Level-2 cannabis misuse was replaced with one of the following baseline measures: treatment group. schizophrenia diagnosis, bipolar disorder diagnosis, substance-induced psychotic disorder diagnosis, psychotic disorder not otherwise specified diagnosis, cocaine misuse diagnosis, amphetamine misuse diagnosis, hallucinogen misuse diagnosis, age, or antipsychotic medication dosage. Separate models were conducted with each variable entered individually as a predictor at Level-2.

Results of these separate models are displayed in Table 2. Results of Model 3 revealed a significant negative effect of schizophrenia diagnosis on the intercept (*p* <. 001), meaning that patients diagnosed with schizophrenia had lower social functioning at baseline compared with other patients. However, schizophrenia diagnosis was not significantly related to the SOFAS change slope (*p* = .315). Results of Model 5 showed that bipolar disorder diagnosis was positively related to the intercept (*p* <. 001), indicating that patients with bipolar disorder had significantly better baseline social functioning compared with other patients. However, bipolar disorder diagnosis was also related negatively to the change slope (*p* = .017), such that patients with bipolar disorder showed less improvement in their social functioning over time than other patients. Finally, results of Model 8 revealed marginally significant effects of cocaine misuse diagnosis on the intercept (*p* = .060) and slope (*p* = .051), which were positive and negative, respectively. Thus, similar to the findings for bipolar disorder, patients diagnosed with cocaine misuse began with somewhat higher levels of social functioning and showed somewhat less change in their social functioning over time.

To assess whether the effect of cannabis misuse on change in social functioning was independent of these possible confounding variables, we ran a final multilevel growth curve model with cannabis misuse plus all other predictors shown in Table 2. Results of this combined model are shown in Table 3. Cannabis misuse was still significantly negatively related to the SOFAS change slope (*p* = .023) even after controlling for all other baseline measures that were (marginally) significantly associated with cannabis misuse. Thus, independent of other baseline characteristics associated with cannabis misuse, patients diagnosed with cannabis disorder showed significantly lower improvement in social functioning over time. No other baseline variables were significantly associated with the SOFAS change slope.

**Table 3:** Results of Combined Multilevel Linear Growth Curve Model Predicting Change in Social Functioning over 30-Months from Cannabis Misuse and other Baseline Measures Simultaneously.

		95% CI		
Predictor / Parameter	Estimate (*SE*)	LL	UL	*p*
Effect on Intercept				
Cannabis misuse (β_01_)	8.78 (3.93)	0.90	16.66	.030
Treatment group (β_02_)	–5.18 (3.38)	–11.96	1.59	.131
Schizophrenia (β_03_)	–16.76 (4.60)	–25.98	–7.53	<.001
MDEP (β_04_)	–9.10 (6.31)	–21.74	3.54	.155
Bipolar disorder (β_05_)	–4.79 (7.70)	–20.22	10.63	.536
SIPD (β_06_)	–23.15 (10.02)	–43.22	–3.08	.025
PDNOS (β_07_)	–10.66 (4.66)	–20.01	–1.32	.026
Cocaine (β_08_)	15.98 (8.37)	–0.79	32.76	.061
Amphetamine (β_09_)	1.27 (5.09)	–8.92	11.46	.804
Hallucinogen (β_010_)	–5.88 (5.59)	–17.07	5.31	.297
Age (β_011_)	–0.73 (0.59)	–1.91	0.46	.224
Antipsychotic medication (β_012_)	–8.10 (6.31)	–20.75	4.54	.205
Effect on Slope				
Cannabis misuse (β_11_)	–3.21 (1.37)	–5.95	–0.47	.023
Treatment group (β_12_)	0.96 (1.18)	–1.40	3.33	.419
Schizophrenia (β_13_)	0.02 (1.56)	–3.11	3.16	.988
MDEP (β_14_)	0.83 (2.09)	–3.36	5.02	.693
Bipolar disorder (β_15_)	–0.62 (2.46)	–5.54	4.30	.801
SIPD (β_16_)	–0.02 (3.28)	–6.59	6.54	.994
PDNOS (β_17_)	0.28 (1.58)	–2.88	3.44	.858
Cocaine (β_18_)	–3.08 (2.66)	–8.41	2.25	.252
Amphetamine (β_19_)	2.01 (1.64)	–1.28	5.30	.227
Hallucinogen (β_110_)	–1.15 (1.86)	–4.88	2.58	.538
Age (β_111_)	0.11 (0.20)	–0.29	0.51	.592
Antipsychotic medication (β_112_)	0.29 (2.03)	–3.79	4.36	.888

*Note*. MDEP = major depressive episode with psychotic features.

SIPD = substance-induced psychotic disorder.

PDNOS = psychotic disorder not otherwise specified.

Approx. *df* = 56.

## Discussion

The profile of our sample, FEP patients with an average age of 20 years, is one of high risk for cannabis misuse. In fact, half of our sample had a cannabis use disorder diagnosed at baseline. Although cannabis misuse was not associated with social functioning at baseline assessment, it was an independent predictor of long-term social functioning. Over a 30-month follow-up, FEP patients without cannabis misuse showed significant improvements in their social functioning, whereas patients who had been diagnosed with cannabis disorder at baseline displayed no such improvement. Cannabis misuse was still significantly associated with social functioning change over the 30-month follow-up even after controlling for all other baseline potential confounders, including pharmacological and psychological treatments.. While patients with and without cannabis disorder showed divergent social functioning trajectories from the first follow-up, the patient groups only differed significantly in their levels of social functioning after 24 months.

In agreement with our results, most recent FEP studies have found a detrimental effect of cannabis misuse on functioning [6–8]. However, our findings are at odds with other studies [10,11]. A possible explanation for this discrepancy is that these studies, assessed patients’ social functioning using a quality of life scale or a scale that integrates symptoms and social functioning. Interestingly, another study from the same group [8] found that the relationship between cannabis and social functioning, when assessed with a quality of life scale, was mediated by symptoms, whereas when assessed in terms of productivity (i.e. being employed or in school) cannabis use at initial assessment emerged as an independent predictor of functional outcome at 1-year and 2-year follow-ups. Similarly, the impact of baseline cannabis misuse on long-term social functioning might be concealed when an overall scale of functioning (which includes symptoms) is used (for example [11].

While the deleterious effects of cannabis on psychosocial functioning and psychopathology have been described in young people from the general population [34], the negative impact on social functioning might be particularly important in young people with recent onset of psychosis, who are known to be prone to psychosocial deterioration. According to the ‘critical period’ hypothesis [35], symptomatic and psychosocial deterioration progresses rapidly during the early phase of psychosis. In the present study, young people with no problematic cannabis use significantly improved their social outcomes over time, whereas those with cannabis misuse did not improve their overall social outcomes. In this sense it may be said that cannabis misuse precludes patients from the benefits expected from specialized FEP services. Consequently, this stresses the need to include specific interventions for the management of cannabis misuse in early intervention services. However, current psychosocial interventions may have limited efficacy in this population [36] and the few available studies of specialized substance abuse treatments for FEP patients have not demonstrated substantially better rates of reduction or abstinence in substance use compared with non-specialized treatments [37,38]. At the same time, it is worth noting that some behavioural treatments for drug abuse in people with severe mental disorders have proven to be efficacious both in reducing substance use and improving community-functioning [39]. Interestingly, Gonzalez-Pinto et al. [12] found that stopping cannabis use after the first psychotic episode contributed to improvement in long-term functional outcomes. In the present study, the subjects who according to the ASSIST scale stopped their problematic cannabis use over the 30-month follow up were only 11 (13.6%) out of 81. The small sample size of this subgroup precludes any reliable analysis.

The means by which cannabis misuse affects social functioning remains to be elucidated. A potential mechanism by which cannabis misuse prevents social improvements might be by impairing some cognitive processes, which have been associated with functional outcomes in FEP [40]. However, recent meta-analyses have demonstrated that patients with schizophrenia and a comorbid cannabis use disorder have the same or lower levels of cognitive impairment as their non-using counterparts [41,42]. Alternatively, cannabis may affect social outcomes by exacerbating of symptoms or other clinical features. Nevertheless, cannabis has only been inconsistently associated with symptoms and other clinical outcomes, other than relapse and treatment non-adherence [5]. In the present study symptom severity or treatment adherence were not associated with social functioning. Therefore, it is likely that other variables not analysed in this study may help to understand the mechanisms underlying the association between cannabis misuse and social functioning. Speculatively, one possible mechanism is intrinsic motivation. While the evidence for an amotivational syndrome due to cannabis use in the general population is conflicting [43], the effects in those patients characteristically affected by motivational deficits (e.g., FEP patients) maybe more pronounced. In support of this idea, intrinsic motivation has been suggested as a decisive mechanism for explaining the relationship between neurocognition and psychosocial functioning in schizophrenia [44].

There are some limitations to this study. First, this study uses a secondary analysis of data from EPISODE-II, a RCT designed to evaluate the effectiveness of an individual and family-based relapse prevention therapy for relapse prevention in clinically remitted FEP patients. Second, the sample size was modest and the influence of variables with small effect sizes might remain undetected. We have reported confidence intervals to show the uncertainty of the results following the recommendations of some statisticians [45]. Third, cannabis misuse was determined by a DSM diagnosis, which arguably can only detect the most severe cases of cannabis misuse. Nevertheless, the percentage of cases detected is not below other first-episodes studies [3]; on the other hand, there was a high degree of consistency between the DSM-IV cannabis misuse disorder diagnosis and the ASSIST-cannabis scale, a valid screening tool for the identification of problematic substance use. Fourth, other variables not examined in the present study, such as specific cognitive dimensions, tobacco use, family support or psychological variables, may also have an impact in social functioning and should be considered in future studies. Finally, the current findings may be restricted to FEP patients after the remission of acute psychotic symptoms receiving care in a specialized first episode service.

On the whole, this study shows that cannabis misuse was highly prevalent (> 50%) within a sample of FEP patients after the remission of acute psychotic symptoms. While an overall improvement in social functioning was observed in this sample, in the context of an early intervention service, those patients with baseline cannabis misuse did not improve their long-term social outcomes during the study period. Therefore, there is a need to develop effective interventions to reduce cannabis misuse and, ultimately, to improve social outcomes, which is an essential goal for specialized services that provide care for young people with psychosis.
